# Phenotypic and Genotypic Analysis of Two Rare Cases of ‐‐ ^SEA^ /‐α^4.3^ HbH Disease

**DOI:** 10.1002/jcla.70379

**Published:** 2026-10-12

**Authors:** Weijia Yang, Xiaoyan Huang, Li Zhang, Xiaoqin Yang, Yaya Guo, Qingping Kang, Min Dou, Guijun Huang

**Affiliations:** ^1^ Department of Eugenics and Genetics Guilin People's Hospital Guilin Guangxi China; ^2^ BGI Genomics co., Ltd. Shenzhen Guangdong China; ^3^ Department of Hepatobiliary Surgery Guilin People's Hospital Guilin Guangxi China; ^4^ Department of Radiology Guilin People's Hospital Guilin Guangxi China

**Keywords:** α‐Thalassemia, HbH disease, phenotype, rare genotype

## Abstract

**Background:**

To report two cases of hemoglobin H (HbH) disease with routine genetic testing missed rare variants, where the phenotype was inconsistent with routine genetic testing results, explore their true genotypes and clinical characteristics, and evaluate the application value of single‐molecule nanopore sequencing (CycloneSEQ platform) in detecting rare thalassemia variants.

**Methods:**

Common thalassemia gene testing was performed on two adult male patients who presented with different complications. The results showed ‐‐^SEA^/αα, which was significantly inconsistent with their hematological parameters. Subsequently, long‐read sequencing of the α‐globin gene cluster was conducted using the CycloneSEQ platform (single‐molecule nanopore sequencing technology), and specific primers were designed based on the results for PCR verification.

**Results:**

The two patients were ultimately diagnosed with rare deletional HbH disease with the genotype *‐‐* ^*SEA*^ */‐α* ^*4.3*^ (NC_000016.10:g.176935‐181274DEL). The deletion range includes the α1 gene and is accompanied by an X‐Y‐Z segment recombination, which is reported here for the first time. Both patients exhibited varying degrees of anemia and complications, suggesting that the same genotype can manifest significant phenotypic heterogeneity.

**Conclusion:**

Conventional thalassemia testing fails to identify rare deletional HbH disease, frequently causing genotype–phenotype discordance and diagnostic bias. The newly reported *‐‐* ^*SEA*^ */‐α* ^*4.3*^ genotype with X‐Y‐Z recombination expands the HbH disease genotypic spectrum and confirms obvious phenotypic heterogeneity. CycloneSEQ nanopore sequencing effectively detects rare complex variants undetectable by routine methods, supporting precise diagnosis of atypical thalassemia with favorable clinical prospects.

## Graphical abstract

We report two unrelated adult patients with rare deletional HbH disease (genotype ‐‐^SEA^/*‐*α^4.3^). This novel 4.3 kb deletion covering the HBA1 gene is accompanied by X‐Y‐Z segment recombination, which was identified by CycloneSEQ long‐read nanopore sequencing. Our findings expand the genotypic spectrum of HbH disease and highlight remarkable phenotypic heterogeneity among patients carrying identical genotypes.

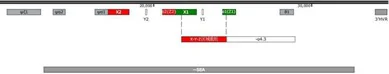

## 1 Introduction

Thalassemia is a group of hereditary autosomal recessive disorders characterized by defective synthesis of hemoglobin (Hb). It involves various molecular defects and is one of the most common genetic diseases globally. The main types include α‐ and β‐thalassemia, caused by mutations in globin genes leading to insufficient production or qualitative changes in the α‐ or β‐globin chains of adult Hb, respectively. Globally, 5%–20% of the population carries one or more α‐thalassemia variants, and approximately 1.5% carries one or more β‐thalassemia variants. Prevalence varies by region, being highest in tropical and subtropical countries, particularly in Southeast Asia and the Mediterranean region [1].

The Guangxi Zhuang Autonomous Region in China is a high‐prevalence area for thalassemia, with a carrier rate as high as 20%–25% [2]. In recent years, with the promotion of third‐generation genetic testing technologies, the detection rate of thalassemia has continued to rise. Zeng et al. [3] reported using multiplex ligation‐dependent probe amplification (MLPA), Sanger sequencing, and single‐molecule real‐time sequencing to identify rare thalassemia genes. Among 55,281 samples in the Guilin area of Guangxi, 16,442 cases of thalassemia were confirmed (detection rate 29.74%), with detection rates for α‐, β‐, and composite α + β thalassemia being 18.57%, 9.99%, and 1.18%, respectively [3].

Currently, there are over 130 known α‐thalassemia variant types (including over 40 deletion types and over 90 non‐deletion types) [4]. Common thalassemia gene testing covers 90%–95% of variant types in the Chinese population. Data show that over 44 types of α‐globin gene variants have been reported in Guangxi, with a detection rate of 91.7% via common thalassemia gene testing. However, approximately 8.3% of rare variants remain missed due to technical limitations [5].

α‐Thalassemia is primarily caused by deletions or mutations in the α‐globin genes. Its clinical presentation varies widely, ranging from asymptomatic carriers to severe anemia and even Hb Bart's hydrops fetalis syndrome. Based on the number of α‐globin gene deletions, it can be classified into silent carrier, trait, HbH disease, and Hb Bart's hydrops fetalis. HbH disease is typically caused by the deletion or dysfunction of three α‐genes, with clinical manifestations ranging from asymptomatic to transfusion‐dependent anemia.

This study reports two unrelated adult males who presented to Guilin People's Hospital in Guangxi, China, with different complications. Common thalassemia testing yielded a result of α‐thalassemia (‐‐^SEA^/αα) and β‐thalassemia (β^N^/β^N^). Due to the inconsistency between the genotype and clinical phenotype, long‐read sequencing was performed using the CycloneSEQ platform (single‐molecule nanopore sequencing). They were ultimately confirmed to have rare deletional HbH disease: ‐‐^SEA^/‐α^4.3^. The ‐α^4.3^ variant (NC_000016.10:g.176935‐181274DEL) is reported here for the first time domestically and internationally.

## 2 Cases Reports

### 2.1 Case 1

Male, 42 years old, from Xing'an County, Guilin, Guangxi.

### 2.2 Clinical History

Admitted to the Department of Gastroenterology on March 29, 2021, due to “recurrent abdominal pain for over half a month.” Previously diagnosed with thalassemia, with hemoglobin fluctuating between 80 and 90 g/L; history of two blood transfusions. Suspected pancreatitis over 10 years ago (details unknown). History of diabetes for 6 years, poorly controlled with oral hypoglycemic agents.

### 2.3 Physical Examination

Pale skin and mucous membranes, mild scleral icterus, liver palpable 3.5 cm below the costal margin, spleen enlarged to 3 cm below the umbilicus, hard texture, and mild tenderness.

### 2.4 Imaging Findings

Abdominal CT showed cavernous transformation of the portal vein due to developmental anomaly, splenomegaly, pancreatic atrophy with calcification, left renal atrophy, right renal cyst, bilateral renal calculi, and paraspinal soft tissue shadows (suggestive of extramedullary hematopoiesis). Gastroscopy and colonoscopy showed chronic atrophic gastritis and chronic colitis.

### 2.5 Hematological Data and Hemoglobin Electrophoresis (Preadmission)

Hb 54 g/L, MCV 74.5 fL, MCH 19.7 pg., HbA 81.1%, HbA_2_ 0.6%, HbH 17.7%, and Hb Bart's 0.6%.

### 2.6 Common Thalassemia Gene Testing

‐‐^SEA^/αα, β^N^/β^N^.

### 2.7 Clinical Management

After multidisciplinary consultation, the patient received small‐volume frequent blood transfusions and partial splenic artery embolization. The condition improved, and the patient was discharged.

### 2.8 Case 2

Male, 48 years old, from Xing'an County, Guilin, Guangxi.

### 2.9 Clinical History

Admitted to the Department of Vascular Surgery on May 10, 2025, due to “swelling of the left lower limb for 1 day.” Diagnosed with thalassemia in childhood; received 1 transfusion at age 11. Received transfusions for splenectomy and cholecystectomy in 2014 (1 unit each). History of diabetes for 5 years, on multiple oral hypoglycemic agents, control status unknown.

### 2.10 Physical Examination

Pitting edema of the left lower limb; no abnormalities in the right lower limb.

### 2.11 Imaging Findings

Echocardiography showed right heart enlargement, moderate tricuspid regurgitation, and pulmonary hypertension. Ultrasound of lower extremity veins showed extensive thrombosis.

### 2.12 Hematological Data and Hemoglobin Electrophoresis (Preadmission)

Hb 129 g/L, MCV 66.8 fL, MCH 18.1 pg., HbA 94.8%, HbA2 0.7%, HbH 4.5%, and Hb Bart's 0.0%.

### 2.13 Common Thalassemia Gene Testing

‐‐^SEA^/αα, β^N^/β^N^.

### 2.14 Clinical Management

Underwent catheter‐directed thrombolysis and percutaneous thrombectomy for lower extremity veins. The condition improved post‐surgery, and the patient was discharged.

## 3 Methods

### 3.1 Ethical Approval

Thalassemia gene testing was performed after obtaining informed consent from subjects. The study was approved by the Medical Ethics Committee of Guilin People's Hospital (2022‐072KY, 2023‐120KY).

### 3.2 Common Thalassemia Gene Testing

Thalassemia gene detection kits from Shenzhen Yilifang Biotechnology Co. Ltd. were used. Gap‐PCR was used to detect 4 types of deletional α‐thalassemia: αα/‐α^3.7^, αα/‐α^4.2^, αα/‐‐^SEA^, αα/‐‐^THAI^. Reverse Dot Blot (RDB‐PCR) was used to detect 3 types of non‐deletional α‐thalassemia (αα/αα^CS^, αα/αα^WS^, and αα/αα^QS^) and 18 types of β‐thalassemia mutations (CD41‐42, CD43, IVS‐II‐654, −28, −29, −30, −32, CD71‐72, βE, CD17, CD31, CD37, CD14‐15, CD27‐28, IVS‐I‐1, IVS‐I‐5, CAP+1, and IntM).

### 3.3 Rare Thalassemia Gene Testing

Long‐read targeted sequencing was performed using the CycloneSEQ platform (single‐molecule nanopore sequencing technology) by BGI Genomics (Shenzhen). This study was approved by the Institutional Review Board of BGI Genomics (IRB24094). The method detects all variant types in the *HBA* and *HBB* genes, including single nucleotide variations (SNVs), deletions, and structural variations (SVs). Specific primers were designed based on the characteristics of *HBA1*, *HBA2*, and *HBB* genes to achieve effective amplification. Multiplex PCR was used to target specific regions of these genes in human peripheral blood genomic DNA, introducing tag sequences for sample identification. Amplification products from multiple samples were mixed, purified, and libraries were constructed by ligating sequencing adapters and barcodes to both ends of DNA fragments. After quality control, sequencing data were aligned with reference sequences. Variants were identified based on alignment and positive judgment criteria to determine the presence and type of α‐ or β‐thalassemia‐associated gene variants.

## 4 Results

### 4.1 Hematological Parameters and Common Thalassemia Testing Results Are Shown in Table 1

**TABLE 1:** Laboratory results from our institution: Case 1 and Case 2.

	Average Hb (g/L)	Admission Hb (g/L)	MCV (fL)	MCH (pg)	HbA (%)	HbA_2_ (%)	HbH (%)	HbBart's (%)	Serum ferritin (ng/mL)	Conventional thalassemia genotypes
Case 1	59.10 ± 13.68	54	74.5	19.7	81.1	0.6	17.7	0.6	954	αα/‐‐^SEA^，β^N^/β^N^
Case 2	122.33 ± 12.42	129	66.8	18.1	94.8	0.7	4.5	0	923	αα/‐‐^SEA^，β^N^/β^N^

*Note:* Average Hb (g/L): Case 1 represents the average of 10 measurements (5 outpatient, 5 inpatient, excluding measurements within 1 month posttransfusion). Case 2 represents the average of 3 measurements (1 outpatient, 2 inpatient).

Hemoglobin electrophoresis results for Case 1 and Case 2 are shown in Figures 1 and 2. The Common Thalassemia Gene Gap‐PCR agarose gel electrophoresis images (Sample 21254‐F for Case 1 in Figure 3 and Sample ZCM for Case 2 in Figure 4) indicated ‐‐^SEA^/αα for both.

**FIGURE 1:**
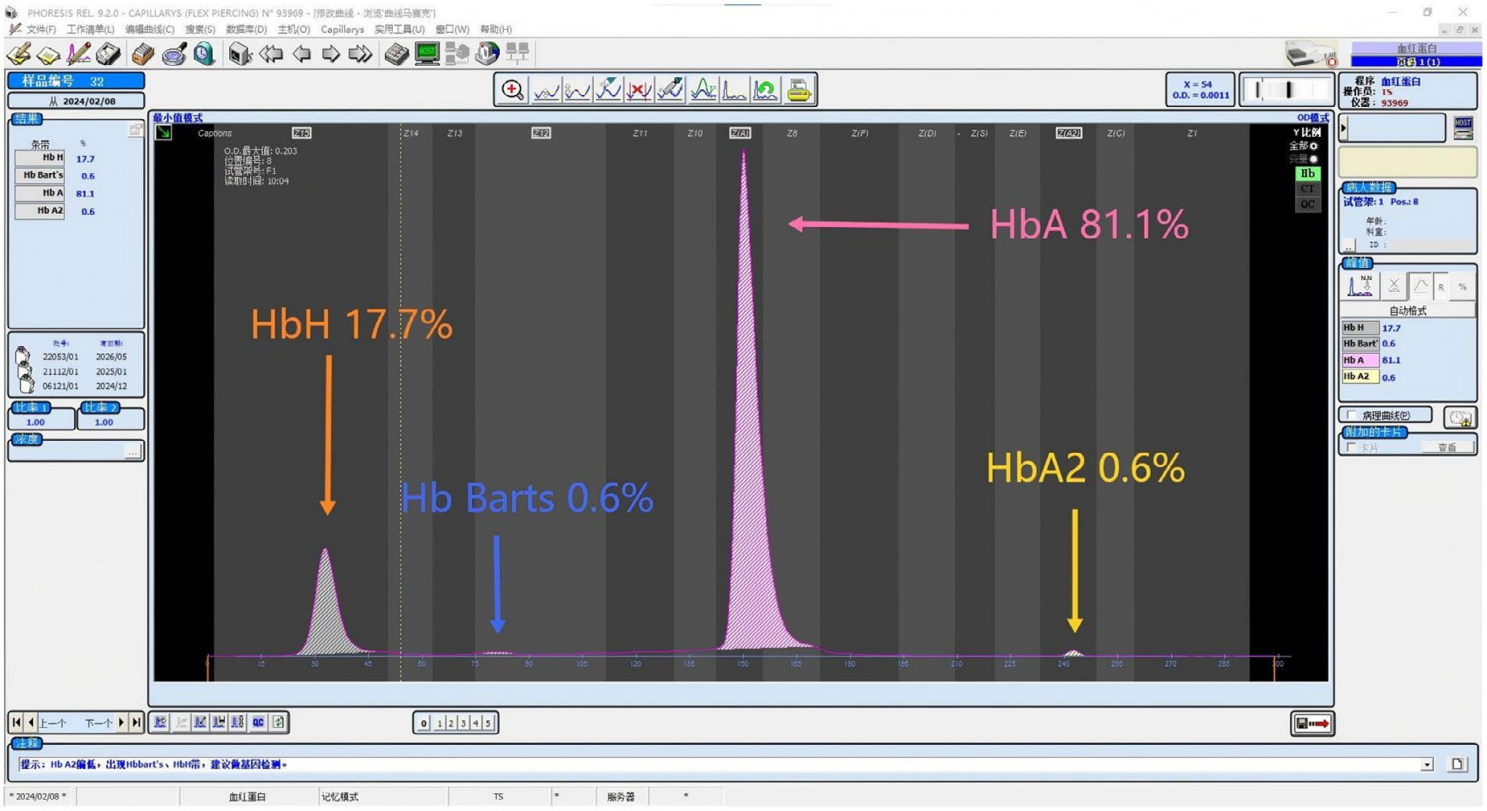
Case 1—Capillary electrophoresis.

**FIGURE 2:**
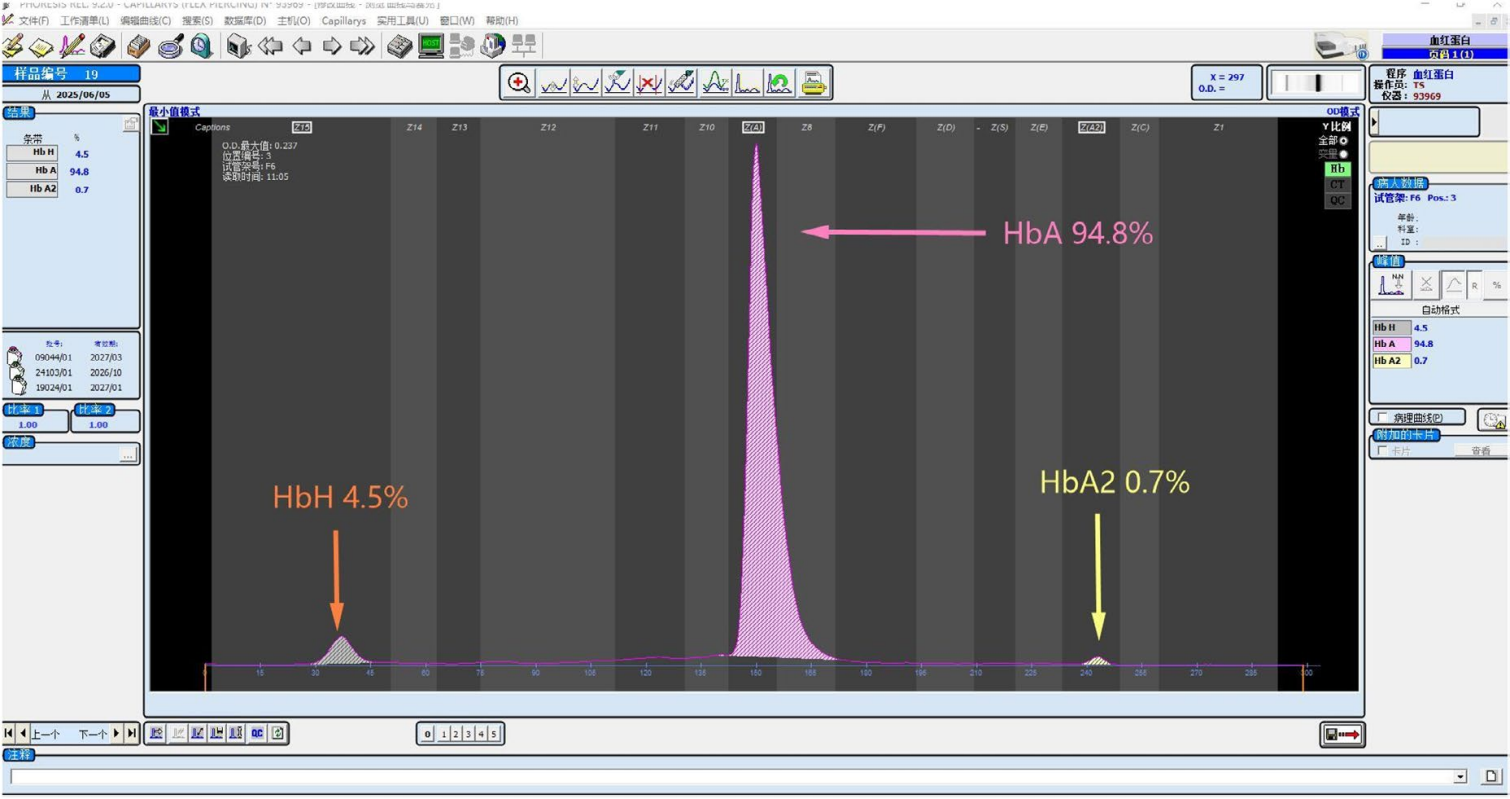
Case 2—Capillary electrophoresis.

**FIGURE 3:**
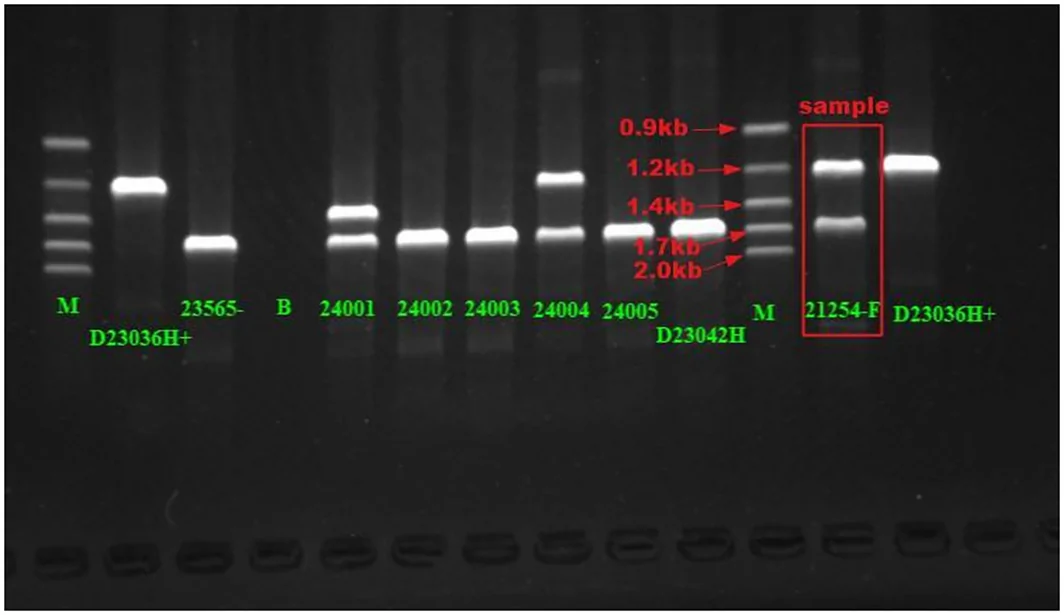
Case 1—Gap‐PCR agarose gel electrophoresis from our institution (21254‐F).

**FIGURE 4:**
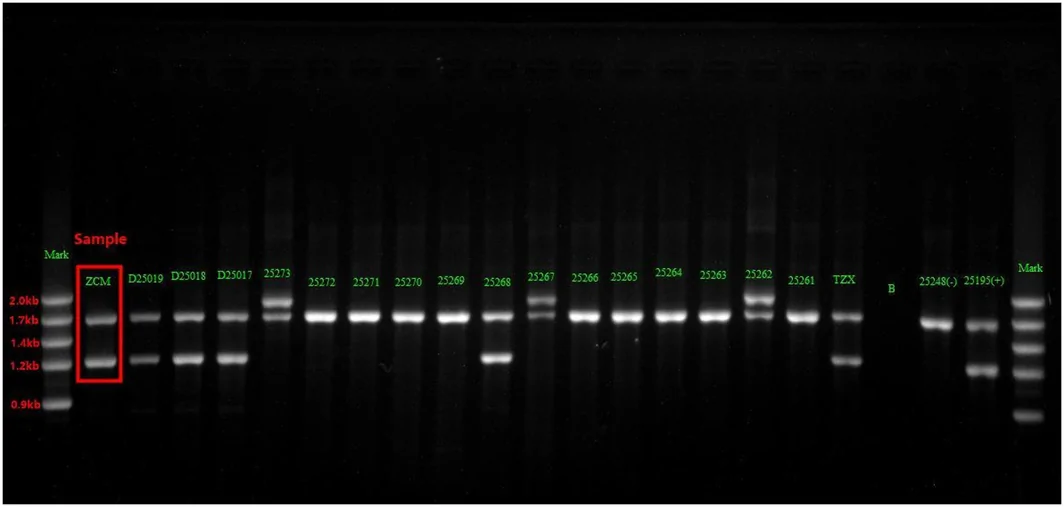
Case 2—Gap‐PCR agarose gel electrophoresis from our institution (ZCM).

### 4.2 Comparison of Complications and Imaging Findings Between Case 1 and Case 2 Is Shown in Table 2

**TABLE 2:** Comparison of complications and imaging findings between Case 1 and Case 2.

Comparison of complications/imaging findings	Case 1	Case 2
Type 2 diabetes	Yes	Yes
Extramedullary hematopoiesis	Yes	No
Portal hypertension	Yes	No
Liver abnormality	Hepatomegaly, intrahepatic bile duct dilation	No
Splenic abnormality	Splenomegaly, splenic cyst, and splenic infarction	Postsplenectomy
Pancreatic abnormality	Atrpphy, calcification, and calculi	No
Renal abnormality	Left renal atrophy, right renal cyst, and bilateral renal calculi	No
Cardiomsgaly	No	Right heart enlargement and pericardial effusion
Pulmonary hypertension	Moderate	Severe
Venous thrombosis	No	Extensive bilateral lower extremities thrombosis

### 4.3 Detection Results From CycloneSEQ Platform (Single‐Molecule Nanopore Sequencing)

Both Case 1 and Case 2 were identified as genotype: ‐‐^SEA^/‐α^4.3^ (NC_000016.10:g.176935‐181274DEL). The ‐α^4.3^ deletion encompasses the α1 gene and is accompanied by an X‐Y‐Z segment recombination (NC_000016.10:g.174111_176934con169854_173130). The schematic of the α‐globin gene cluster and variants is shown in Figure 5. Based on these results, specific primers were designed for PCR amplification and verification. Agarose gel electrophoresis confirmed the PCR product fragment sizes matched the expected design, as shown in Figure 6.

**FIGURE 5:**
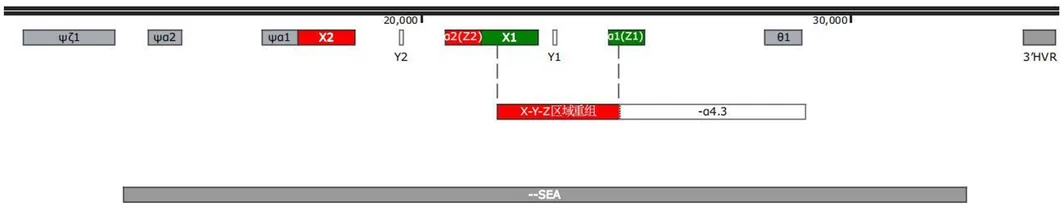
Schematic diagram of the α‐globin gene cluster (showing the ‐α^4.3^ deletion and X‐Y‐Z segment recombination).

**FIGURE 6:**
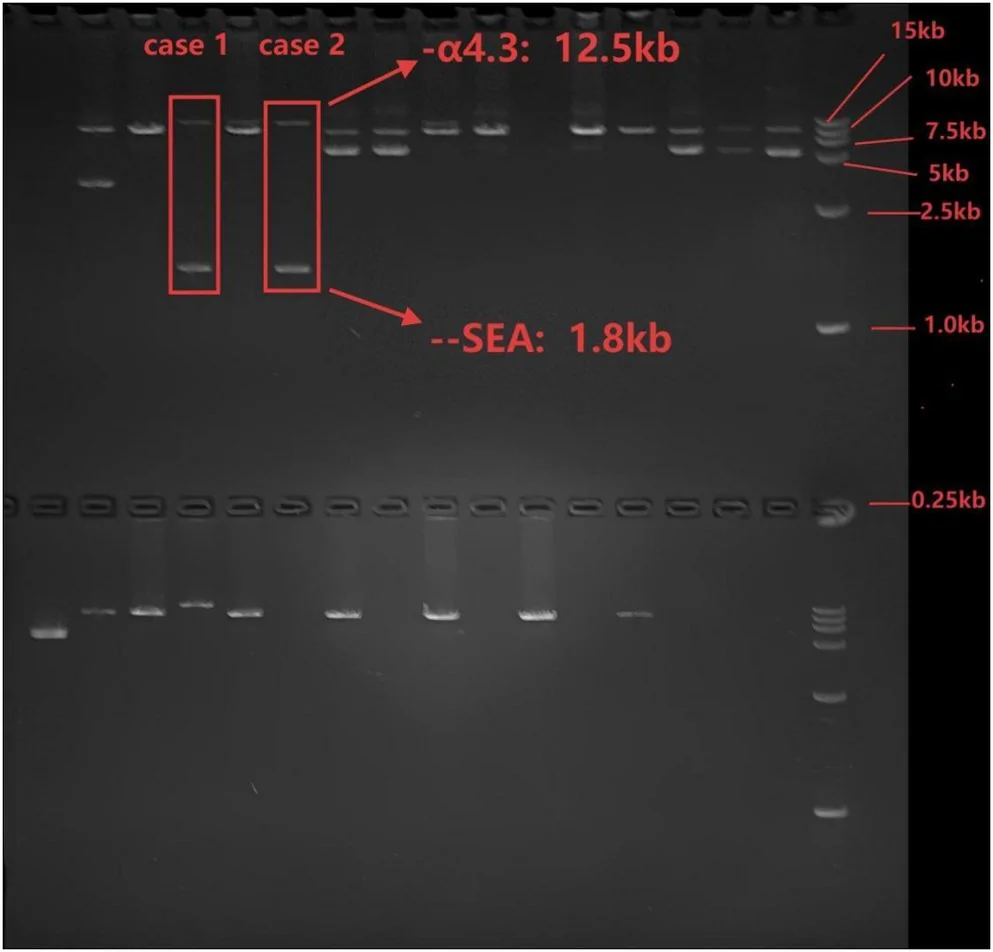
Agarose gel electrophoresis image of targeted amplification products.

## 5 Discussion

### 5.1 Molecular Mechanism and Clinical Characteristics of HbH Disease

Hemoglobin H (HbH) disease is a form of thalassemia intermedia caused by a significant reduction in α‐globin chain synthesis. Normally, individuals possess four functional α‐globin genes (αα/αα). When three of these genes are deleted or inactivated, the single remaining α‐gene is insufficient to maintain the balance between α‐ and β‐globin chains. This leads to the formation of β‐tetramers (HbH) from excess β‐chains, causing hemolytic anemia and related complications. Furthermore, both HbH and Hb Bart's have high oxygen affinity and lack heme–heme interaction, preventing effective oxygen transport and release to tissues, leading to tissue hypoxia [1].

HbH disease is classified into deletional and non‐deletional types. Prevalence reports in China vary among scholars. Zhang et al. [6] reported that among 283 confirmed HbH cases in Guangdong, deletional HbH accounted for the majority (202 cases, 71.38%), while non‐deletional HbH accounted for 81 cases (28.62%). Li et al. [7] analyzed 9555 individuals in Guangzhou, finding 64 cases of HbH disease, with an HbH band detection rate of 75.00%. The most common genotypes were ‐‐^SEA^/‐α^3.7^ (56.26%) and ‐‐^SEA^/‐α^4.2^ (39.24%). Pang et al. [8] reported 678 genotyped HbH cases, with 505 (74.5%) non‐deletional and 173 (25.5%) deletional cases, including 56 cases of ‐‐^SEA^/‐α^4.2^. These discrepancies may be related to sample sources. However, all scholars report that patients with non‐deletional HbH disease exhibit more severe anemia than those with deletional types [1, 6, 7, 8, 9]. This may be because non‐deletional mutations affect α‐globin chain structure or synthesis by interfering with mRNA processing/translation or altering protein stability and interactions. It has also been reported that non‐deletional mutations are mostly associated with key genomic regions for normal α‐globin gene expression, involving oligonucleotide insertions and deletions [10, 11, 12]. Most cases of deletional HbH disease are often overlooked because they are not transfusion‐dependent, and patients are typically diagnosed only when they present with severe infections or complications, as observed in the two cases reported here.

### 5.2 Interpretation of the New Variant Coordinates

The ‐α^4.3^ variant (NC_000016.10:g.176935‐181274DEL) is a novel rare deletion reported for the first time in our study [13]. This deletion region covers the α1 globin gene but does not involve the α2 gene. The deletion begins at exon 2 of the α1 gene (range: NC_000016.10:g.176935‐181274) and is accompanied by a recombination event where the sequence NC_000016.10:g.174111_176934 is replaced by the sequence NC_000016.10:g.169854_173130 (denoted as NC_000016.10:g.174111_176934con169854_173130).

### 5.3 Possible Mechanisms of Phenotypic Heterogeneity

Both patients reported in this study are adult males with the identical genotype (‐‐^SEA^/‐α^4.3^), yet their clinical presentations differ significantly. Case 1 presented with moderate‐to‐severe anemia (Mean Hb 59.10 ± 13.68 g/L), high HbH (17.7%), and multi‐organ involvement including hepatomegaly, portal hypertension, and pancreatic atrophy. In contrast, Case 2 had near‐normal hemoglobin (Mean Hb 122.33 ± 12.42 g/L), low HbH (4.5%), and presented mainly with thrombotic disease and pulmonary hypertension. The underlying reasons for this discrepancy are analyzed below.

#### 5.3.1 Comparative Analysis of Hematological Parameters

Since the size of the ‐α4.3 deletion fragment is similar to that of the ‐α4.2 deletion, theoretically, the phenotypes of ‐‐SEA/‐α4.2 and ‐‐SEA/‐α4.3 should be comparable. We compiled the complete blood count and hemoglobin electrophoresis results from 11 adult cases identified as ‐‐SEA/‐α4.2 (without concurrent β‐thalassemia) by hemoglobin electrophoresis and genotyping in our laboratory over the past 5 years, as shown in Table 3. The mean Hb was 97.18 ± 14.77 g/L and HbH was 5.88 ± 4.45 (%). The Hb results are close to the 95.33 ± 14.56 g/L reported by Li [7], and the HbH band detection rate is also similar to the 75% reported by the same author. The Hb results are close to 96.69 ± 15.72 g/L reported by Zhang et al. [6] (including both ‐‐SEA/‐α4.2 and ‐‐SEA/‐α3.7 7 deletion types), and the HbH results are also close to their 7.00 ± 4.18 (%). The Hb results are close to 91.4 ± 11.6 g/L reported by Pang et al. [8] (including three deletion types: ‐‐SEA/‐α4.2, ‐‐SEA/‐α3.7, and ‐‐Thai/‐α3.7), and the HbH results are also close to their 6.5 ± 3.1 (%). Case 2's Hb of 129 g/L is significantly higher than the statistical results for deletion‐type HbH disease from our laboratory and the aforementioned literature, while the HbH result is close to those reported by our laboratory and the literature. Case 1's Hb of 54 g/L is significantly lower than the statistical results for deletion‐type HbH disease from our laboratory and the aforementioned literature. Case 1's Hb and HbH values are relatively closer to results for non‐deletion‐type HbH disease. Zhang et al. [6] reported non‐deletion‐type HbH disease with Hb 78.69 ± 15.65 g/L and HbH 15.65 ± 6.18 (%), and Pang et al. [8] reported non‐deletion‐type HbH disease with Hb 80.3 ± 13.6 g/L and HbH 11.7 ± 4.0 (%).

**TABLE 3:** Hematological parameters of 11 cases with ^‐‐SEA^/‐α^4.2^ in our hospital.

No.	Sex	Age (years)	Hbe (g/L)	MCVe (fl)	MCHe (pg)	HbAe (%)	HbA2e (%)	HbFe (%)	HbHe (%)	HbBart'se (%)
1	Female	28	100	72.7	22.3	97.3	2.3	0.4	0	0
2	Female	28	101	71.7	23.7	96.7	2.5	0.8	0	0
3	Male	36	117	62.4	17.9	93.6	1.2	0	5.2	0
4	Female	30	112	68.5	21.4	97	2.6	0.4	0	0
5	Female	31	82	64.5	18	91.1	1	0	7.5	0.4
6	Female	45	83	66.4	19.1	88.4	0.8	0	10.8	0
7	Female	38	74	60.9	17.7	93.1	1.1	0	5.3	0.5
8	Female	21	82	66.7	18.3	90	0.7	0	9.3	0
9	Male	31	107	56.1	18.8	87.9	0.8	0	10.2	1.1
10	Male	23	113	53.6	17.4	93.3	1	0	4.6	0
11	Male	48	98	68.2	19.3	87.2	1	0	11.8	0
*X*		32.63	97.18	64.7	19.45	92.33	1.36	0.15	5.88	0.18
SD		8.44	14.77	6.03	2.09	3.70	0.73	0.27	4.45	0.35

#### 5.3.2 Exploring the Molecular Mechanisms of HbH Disease With Identical Genotypes but Different Phenotypes

It is generally accepted that the α2‐globin gene produces approximately 2–3 times more α‐globin mRNA than the α1‐globin gene. In the two novel deletion‐type mutations in this study, the deletions occur in the α1‐globin gene. Theoretically, the phenotype should not be too severe, meaning that the phenotypes of Case 1 and Case 2 should be similar to HbH disease with the genotype ‐‐SEA/‐α4.2. Clearly, Case 1 shows a substantial discrepancy.

##### 5.3.2.1 Economic Status and Living Environment

The two patients differ significantly in economic circumstances. Case 1 came from an impoverished family. After graduating from middle school (around age 16), he left to work elsewhere, primarily in house renovation. His work involved approximately 10 h of physical labor daily, including carrying heavy renovation materials such as sand and cement weighing dozens of pounds, followed by tile laying and wall plastering. In recent years, his physical strength has noticeably declined, but at the time of consultation, he was still performing wall plastering work with relatively low income. Years of physical labor, irregular diet, and poor sleep patterns resulted in frequent abdominal pain and an emaciated physique in Case 1. In contrast, Case 2 comes from a relatively affluent family. As an adult, he works in music, rarely engages in outdoor activities, and primarily performs indoor mental labor centered on music editing, with a middle‐income level. From a pathophysiological perspective, HbH inclusion bodies make red cell membranes more fragile, and oxidative stress accelerates the destruction of abnormal red cells. Long‐term high‐intensity physical labor increases systemic oxygen consumption and oxidative stress levels, exacerbating extravascular hemolysis. In HbH disease, erythroid compensatory proliferation is vigorous, and folate requirements are significantly higher than in normal individuals. Chronic dietary irregularity easily leads to insufficient folate intake, directly limiting the bone marrow's compensatory hematopoietic capacity. Therefore, although Case 1 could not provide medical records from before age 35, we can reasonably hypothesize that his initial hemoglobin level was likely around 90 g/L. Due to long‐term overwork, lack of regular rest days, and chronic bodily stress from nutritional deficiency, combined with the patient's limited economic resources and education level leading to neglect of his progressive physical deterioration, various compounding factors on the basis of HbH disease gradually worsened his condition, resulting in successive complications: hepatomegaly, intrahepatic bile duct dilation, portal hypertension, splenic abscess, splenic necrosis, pancreatic atrophy, left renal atrophy, bilateral renal calculi, etc. These further weakened bone marrow compensatory efficiency. Due to limited financial resources, he was unable to receive standardized, systematic treatment, resulting in long‐term maintenance of Hb at relatively low levels. A similar explanation is provided by Lal et al. [14], who noted that “in practice, environmental factors (recurrent infections, malaria, or nutrition) may alter the phenotypic expression of HbH disease”. Other scholars have proposed that the heterogeneity of HbH disease stems from the complex interactions among genetic variation profiles, gene co‐segregation effects, and environmental factors, making the relationship between genotype and phenotype a continuous, gradually changing trend. This is essentially a dynamic process involving the combined action of genetic and environmental factors [15, 16].

##### 5.3.2.2 Splenectomy Improved Hb Levels in Case 2

In a multicenter retrospective study of 1421 HbH disease patients in China, Wu et al. [17] reported that splenectomy can increase hemoglobin levels by 10–30 g/L in HbH disease patients and reduce transfusion requirements. The spleen is the primary organ for extravascular destruction of abnormal red blood cells containing HbH inclusion bodies. Viprakasit et al. [11] noted that splenectomy removes the sequestering effect of splenic phagocytosis, thereby prolonging the survival time of abnormal red cells in the peripheral circulation and consequently raising hemoglobin levels. However, this surgery does not correct the underlying globin gene defect, and HbH inclusion bodies continue to persist within red blood cells.

Additionally, due to the instability of HbH tetramers and peripheral hemolysis caused by red cell precipitation, accompanied by endothelial injury, platelet activation, and alterations in coagulation regulatory proteins, these factors collectively contribute to a hypercoagulable state. Adult patients who have undergone splenectomy and have inadequate transfusion are at higher risk for thrombosis and pulmonary hypertension [11]. This is consistent with the complications observed in Case 2. Further inquiry into the medical history revealed that Case 2 had rarely used aspirin for thrombosis prophylaxis after splenectomy. Therefore, although Case 2's Hb level approaches that of normal males, the occurrence of severe venous thrombosis in middle age is consistent with disease progression, attributable to the combined effects of HbH disease and splenectomy.

##### 5.3.2.3 The Influence of Modifier Genes

A recent large‐scale cohort study by Liu Yumeng, “A Cohort Study of Heterogeneity in 591 HbH Disease Patients,” found significant variation in clinical severity among HbH disease patients with identical genotypes. Furthermore, genotype–phenotype association analysis revealed that rs150683770, located on the FAM234A gene on chromosome 16, is the leading SNP affecting the phenotypic severity of HbH disease and is also significantly associated with elevated TRF (transferrin) levels. Association analysis also identified rs76889456, located on the NPRL3 gene on chromosome 16, as significantly associated with decreased TBIL (total bilirubin) levels. The study also identified three leading SNPs significantly associated with HbH band content, located on the FAM234A and AXIN1 genes on chromosome 16, and the PPP5DIP gene on chromosome 19. Significant signals were also detected for rare variants in both coding and noncoding regions of the FAM234A and AXIN1 genes. Among the 591 HbH cases in this study, 224 were deletion types, of which 3 cases (genotype ‐‐^SEA^/‐α^3.7^) had severe clinical phenotypes, presenting with severe growth and developmental delay at ages 4–5. Database queries by the author showed that rs150683770 has the highest frequency in the Guangxi population, exceeding the 0.01 frequency in Guangdong [18]. This study represents a major breakthrough in explaining the molecular mechanisms of phenotypic heterogeneity in HbH disease and suggests possible molecular mechanisms for HbH disease with identical genotypes but different phenotypes.

The above represents a hypothetical explanation of the phenotypic differences between the two cases based on clinical and laboratory findings, combined with literature reports, in the absence of α‐thalassemia modifier gene testing.

## 6 Early Precision Diagnosis and Standardized Management of HbH Disease

In this study, due to the inconsistency between genotype and phenotype in two unrelated individuals, single‐molecule nanopore sequencing was used to further investigate the etiology. We ultimately confirmed the genotype and reported the ‐‐^SEA^/‐α^4.3^ (NC_000016.10:g.176935‐181274DEL) deletional HbH disease for the first time among rare variants. This highlights the necessity of further investigation into similar “genotype–phenotype mismatch” cases, particularly given the highly heterogeneous nature of HbH disease. If Case 1 and Case 2 had been diagnosed early (e.g., in early childhood or adolescence) and managed with close monitoring, complications such as diabetes, hepatobiliary issues, pulmonary hypertension, and venous thrombosis could potentially have been prevented, significantly improving quality of life in middle and later age. Without early correct diagnosis and standardized treatment, HbH patients may experience age‐related complications throughout their lives, requiring intervention from a multidisciplinary team. Future research should combine genome‐wide association studies (GWAS) and pedigree studies to deeply explore modifier gene networks and their interactions with environmental factors.

## Funding

This work was supported by Open Project of NHC Key Laboratory of Thalassemia Medicine (Project Title: Precise Diagnosis and Intervention Strategies for Thalassemia with Genotype–Phenotype Inconsistency; Grant Number: GJWJWDP202303); and Self‐funded Project of Western Medicine Category, Health Commission of Guangxi Zhuang Autonomous Region (Project Title: Genotyping Study of Silent and Rare Variants of Thalassemia in Couples Undergoing Pregnancy; Grant Number: Z‐C20221640).

## Ethics Statement

The study was conducted in accordance with the Declaration of Helsinki (as was revised in 2013). The study was approved by the Ethics Committee of the Guilin People's Hospital (Approval number: 2023‐120KY, 2022‐072KY).

## Consent

Patient consent was obtained for publication of this study.

## Conflicts of Interest

We confirm that all authors of this manuscript have reviewed and consented to its submission and publication.

## Data Availability

All data generated or analyzed during this study are included in this article. Further enquiries can be directed to the corresponding author.
